# Intratumor Heterogeneity of MIF Expression Correlates With Extramedullary Involvement of Multiple Myeloma

**DOI:** 10.3389/fonc.2021.694331

**Published:** 2021-06-29

**Authors:** Juan Xu, Nanhui Yu, Pan Zhao, Fangfang Wang, Jingcao Huang, Yushan Cui, Hong Ding, Yan Yang, Yuhan Gao, Ling Pan, Hong Chang, Yu Wu, Bing Xiang, Yuping Gong, Xiao Shuai, Li Hou, Liping Xie, Ting Niu, Ting Liu, Li Zhang, Weiping Liu, Wenyan Zhang, Ying Qu, Wei Lin, Yimin Zhu, Sha Zhao, Yuhuan Zheng

**Affiliations:** ^1^ Department of Hematology, Institute of Hematology, West China Hospital, Sichuan University, Chengdu, China; ^2^ Department of Anesthesiology, The Second Xiangya Hospital of Central South University, Changsha, China; ^3^ Hunan Provincial Key Lab of Emergency and Critical Care, Hunan Provincial People’s Hospital, Changsha, China; ^4^ Department of Hematology, Affiliated Hospital of North Sichuan Medical College, Nanchong, China; ^5^ Department of Pathology, West China Hospital, Sichuan University, Chengdu, China; ^6^ State Key Laboratory for Oncogenes and Related Genes, Division of Cardiology, Renji Hospital, School of Medicine, Shanghai Cancer Institute, Shanghai Jiaotong University, Shanghai, China

**Keywords:** extramedullary multiple myeloma, MIF, immunohistochemistry staining, xenograft models, single-cell sequencing

## Abstract

Macrophage migration inhibitory factor (MIF) has been shown to promote disease progression in many malignancies, including multiple myeloma (MM). We previously reported that MIF regulates MM bone marrow homing and knockdown of MIF favors the extramedullary myeloma formation in mice. Here, based on MIF immunostaining of myeloma cells in paired intramedullary and extramedullary biopsies from 17 patients, we found lower MIF intensity in extramedullary MM (EMM) versus intramedullary MM (IMM). Flow cytometry and histology analysis in xenograft models showed a portion of inoculated human MM cells lost their MIF expression (MIF^Low^) *in vivo*. Of note, IMM had dominantly MIF^High^ cells, while EMM showed a significantly increased ratio of MIF^Low^ cells. Furthermore, we harvested the extramedullary human MM cells from a mouse and generated single-cell transcriptomic data. The developmental trajectories of MM cells from the MIF^High^ to MIF^Low^ state were indicated. The MIF^High^ cells featured higher proliferation. The MIF^Low^ ones were more quiescent and harbored abundant ribosomal protein genes. Our findings identified *in vivo* differential regulation of MIF expression in MM and suggested a potential pathogenic role of MIF in the extramedullary spread of disease.

## Introduction

The proliferation of clonal plasma cells in multiple myeloma (MM) is typically confined to the bone marrow (BM). However, the extramedullary spread of MM cells, defined as extramedullary multiple myeloma (EMM), may occur at any time in the course of MM (1). Patients with EMM have inferior outcomes (2, 3). Broadly speaking, there are two main types of EMM: plasmacytomas extending from local bone lesions (para-skeletal or extramedullary bone-related, EM-B) or resulting from hematogenous dissemination (extra-skeletal or extramedullary-extraosseous, EM-E) (1, 4, 5). Previous studies have suggested that EM-E showed an even worse prognosis than EM-B (3). In addition, plasma cell leukemia (PCL) might be considered as a specific variant of EMM (5). Nevertheless, some authors have suggested that PCL should be excluded from the EMM spectrum because of PCL’s unique disease entity (4).

The pathogenesis of EMM has not been fully understood. Various genetic and microenvironment mechanisms might affect the occurrence of EMM, including the presence of 13q14 deletion, t(4;14) or TP53 mutation, frequent mutations in the RAS pathway (6–10), the impairment of C–X–C motif chemokine receptor 4 (CXCR4)/stromal-derived factor 1Alpha(SDF-1α) (11–13), differential expression of adhesion molecules, such as very-late-antigen-4 (VLA-4), CD56, CD44 and P-selection (14–17), and upregulation of angiogenic factors like CD31 and endoglin (16). We previously identified macrophage migration inhibitory factor (MIF), a soluble pro-inflammatory cytokine, as a regulator of MM BM homing (18). MIF is highly expressed in MM BM and regulated downstream adhesion molecules’ expression in MM cells; knocking down MIF in MM cells hampered MM adhesion to BM stromal cells *in vitro*, therefore resulting more extramedullary tumors in an SCID mice model (18). To investigate the variation of MIF in EMM versus the parental intramedullary MM (IMM), we initiated this comparative study using paired BM and extramedullary biopsies from a series of MM patients. MIF regulation and the associated single-cell trajectories in EMM were further studied using xenograft mouse models.

## Materials and Methods

### Patient Samples

Formalin-fixed, paraffin-embedded (FFPE) paired tumor samples of BM and extramedullary tissues were retrospectively collected from 17 MM patients who had extramedullary involvement with the following inclusion criteria: 1) the patient was admitted to our hospital from 2014 to 2019; 2) the patient with confirmed MM had pathologically verified extramedullary involvement either at initial diagnosis (primary EMM) or during relapse/progression (secondary EMM); 3) imaging or physical examination showed measurable lesions of EMM. Both EM-B (extramedullary bone-related) and EM-E (extramedullary-extraosseous) myeloma were included in this study; 4) EMM and the corresponding IMM samples from each patient were collected at the same disease stage. Patients with solitary plasmacytoma, PCL, lymphoma with plasmacytic differentiation, or any additional malignancy were excluded. Cytogenetic examination of BM plasma cells was recommended by physicians. This study was approved by the Ethical Committee of West China Hospital, Sichuan University, China. All patients were consented for use of their medical records and samples for research.

### Patient-Derived Primary MM Cells

With the approval by the Ethical Committee of West China Hospital, primary MM cells were obtained from consenting patient #16 when MM progressed with extensive extramedullary disease. Mono-nuclear cells (MNCs) were separated from his iliac BM aspirate by Ficoll-density gradient centrifugation. CD138+ myeloma cells were flow-sorted (Beckman Coulter CytoFLEX platform) using phycoerythrin (PE)-conjugated mouse anti-human CD138 antibody (BD, Biosciences, Cat.# 552026), and cryopreserved in our department tissue bank. For inoculation, the primary MM cells were resuscitated, cultured for 24 h in RPMI-1640 medium with 10% fetal bovine serum (FBS) in 5% CO2 atmosphere at 37°C, washed with phosphate buffered saline (PBS), and then resuspended in PBS at a concentration of 5 × 10^6^/ml.

### Human MM Cell Line and CRISPR Based Target Gene Knock Out

The human MM cell line ARD was a generous gift from Prof. Yiguo Hu (State Key Laboratory of Biotherapy and Cancer Center, West China Hospital, Sichuan University). ARD cells were authenticated by short tandem repeat (STR) profiling (GENEWIZ, Inc. Suzhou, China) before experiment. We knocked out MIF gene in the ARD cells by the CRISPR/Cas9 system as previously described (19). The plasmid pHKO23 was also kindly gifted from Prof. Yiguo Hu. The single guided RNAs (sgRNAs) targeting human MIF gene were as follows:

CACCGAGCTCGGAGAGGAACCCGTC(fwd); AAACGACGGGTTCCTCTCCGAGCTC (rev).

Both ARD and MIF^−/−^ ARD cells were stably infected by luciferase expressing lentivirus (HanBio Inc., Shanghai, China). All cells were cultured in RPMI-1640 medium supplemented with 10% fetal bovine serum, 100 U/ml penicillin, and 100 g/ml streptomycin at 37°C and 5%CO_2_, and were mycoplasma free. Cells were resuspended in PBS at 2 × 10^7^/ml for injection.

### *In Vivo* Mouse Models of Human MM

All animal experiments were approved by the Animal Care and Use Committees of West China Hospital, Sichuan University and were performed in accordance with the ethical standards. To establish xenograft models, patient-derived primary MM cells or luciferase-expressing ARD cell line were implanted into six-week-old female immunocompromised B-NDG mice (NOD-*Prkdc^scid^ IL2rg^tm1^*/Bcgen, Biocytogen Jiangsu CO., Ltd.) *via* tail vein injection. A total of 13 mice were used in this study, including those challenged with the primary MM cells (0.5 × 10^6^ cells per mouse, n = 4) or wild type (w.t.) ARD cells (2 × 10^6^ cells per mouse, n = 5), and control mice given MIF^−/−^ ARD cells (2 × 10^6^ cells per mouse, n = 2) or vehicle (100 μl PBS, n = 2). Mice were housed in pathogen-free conditions and inspected at least twice a week to monitor general health and disease symptoms. Mice in morbid state were sacrificed. Eyeballs were removed under anesthesia to collect blood. Subsequently, they were euthanized *via* cervical dislocation and dissected for further examinations.

### *In Vivo* Bioluminescent Imaging

For tumor monitoring, cell line-derived MM bearing mice were subjected to *in vivo* bioluminescent imaging (BLI) using IVIS spectrum (PerkinElmer, Inc.), on days 7, 14, 21, 28 post inoculation. D-luciferin, potassium salt (Biovision, USA, Cat.# 7903), a substrate of luciferase, was injected intraperitoneally (15 mg/ml in PBS, with 150 μl per mouse). At 15 min after administration, image acquisition was performed under isoflurane anesthesia.

### Immunohistochemistry Analysis and Result Quantification

Tumor specimens were fixed in 10% neutral buffered formalin, decalcified (bone tissues), paraffin-embedded and then processed for immunohistochemistry (IHC) staining. FFPE sections were deparaffinized and rehydrated, and incubated with 3% H_2_O_2_ for 10 min to block endogenous peroxidase activity. Antigens were retrieved with sodium citrate buffer (10 mM, pH 6.0, at 98°C for 10 min). Nonspecific binding was blocked with 5% normal goat serum in TBST, for 1 h at room temperature. Primary antibodies including rabbit polyclonal MIF antibody (dilution 1:400, Santa Cruz Biotechnology, Cat.# FL-115) and rabbit monoclonal CD138 antibody (dilution 1:400, Abcam, Cat.# ab128936) were applied overnight at 4°C. Then, the sections were incubated with biotinylated goat anti-rabbit IgG and streptavidin–biotin complex (SABC) following manufacturer’s instructions (Boster Biological Technology, Cat.# SA1022, China). Signal was developed by diaminobenzidine (DAB) detection kit (ZSGB-Bio, China, Cat.# ZLI-9031), followed by hematoxylin re-dying. The stained slides were imaged under an upright fluorescence microscope (AX10 imager A2/AX10 cam HRC, Zeiss, Germany). For better evaluating MIF intensity, the paired IMM and EMM samples were prepared and photographed for MIF staining synchronously. To evaluate DAB intensity of MIF staining in patients’ slides, wide-field images were captured by the TissueFAXS system (TissueGnostics) as previously described (20), and the mean MIF intensity was quantified using the StrataQuest software (TissueGnostics) followed the manufacturer’s instruction. Pathological analyses of all slides were reviewed by an expert pathologist.

### Opal Multicolor Staining

We used opal multicolor staining to visualize CD138 and MIF on selected human FFPE slides with Opal 4-color manual IHC kit (PerkinElmer, Inc.) as previously described (21).

### Flow Cytometry Analysis

MM cells engraftment in different murine organs was assessed by flow cytometry as previously described using Beckman Coulter CytoFLEX platform (18). Single-cell suspensions were prepared from blood, spleen, liver, lung, kidney and femurs (BM) following relevant guidelines (22). Collagenase D (Sigma-Aldrich, Cat.# 11088858001) was used (1 mg/ml, at 37°C for 30 min) for enzymatic digestion of solid organs, like lung and kidney. Myeloma cells were identified by phycoerythrin (PE)-conjugated mouse anti-human CD138 antibody (BD, Biosciences, Cat.# 552026). Since MIF was an intracellular protein, cells were fixed and permeabilized using the intracellular Fix & Perm buffer set (Invitrogen eBioscience, Cat.# 88-8824-00), followed by MIF staining with Alexa Fluor 488-conjugated mouse anti-human MIF antibody (Clone# 932606) (R&D systems, Cat.# IC2891G). 7-Aminoactinomycin D (7-AAD) (AAT Bioquest, Cat.# 17501) was applied to label non-viable cells before permeabilization. The results were analyzed by the FlowJo software.

### Single Cell Sequencing and Data Analysis

Splenic cells were collected from a tumor-bearing mouse xenografted with ARD cells. CD138-positive MM cells were then sorted by PE-conjugated CD138 antibody (BD, Biosciences, Cat.# 552026) using flow cytometry technology. The construction, sequencing and processing of the single-cell transcriptome library was conducted by the Chengdu Neo-life Hope Medical Laboratory Co. Ltd.

MacoskoDropSeq pipeline was used to generate the single-cell transcriptome profile (23). The raw pair-end sequencing data were first processed using DropSeq Core computation protocol developed at McCarrll’s lab. The derived digital expression matrix was then used to generate Seurat object using Seurat R package. The downstream analyses and data visualization were performed using Seurat functions. The following Seurat functions were used in the Seurat pre-processing pipeline: NormalizeData, ScaleData were used for calculate the comparable expression values; FindVariableFeatures were used to include the variable genes that contribute to the overall similarity/variability of cellular transcriptomic profiles; RunPCA, FindNeighbors, FindClusters, RunTSNE, and RunUMAP were used to calculate the dimension-reduction coordinates for visualization and to perform unsupervised clustering. In the downstream analyses, we used Uniform Manifold Approximation and Projection (UMAP) coordinates to visualize the layout of the cells. We used Monocle2 software to explore the possible differentiation path of these xenografted plasma cells and identify the subclusters using the default software parameters (24).

We used top 10 principle components to represent the digital expression profiles of each cells, and calculate the median values of these 10 principle components of each putative clusters. Then we calculate the overall dissimilarity using the cosine distances algorithm. The abovementioned dissimilarity measure was defined in TooManyCells method (25). The hierarchical cluster was plotted based on the dissimilarity matrix of each cluster.

### Statistical Analysis

Statistical analyses were performed using Graphpad Prism 8 (GraphPad Softwares, USA). Comparisons of continuous variables were made by student’s *t*-test or analysis of variance (ANOVA). The co-expression relationship between two genes was tested by simple linear regression. Mann–Whitney U test was used to compare variables with no specific distribution from independent samples. A *p <*0.05 was considered statistically significant.

## Results

### Differential MIF Expression in EMM Versus IMM in Patients

Previously, we showed that MIF-knockdown (MIF-KD) MM cells had decreased BM homing and were likely to form extramedullary tumors in MM mouse models (18). In this study, we retrospectively collected FFPE biopsies of IMM and EMM from 17 MM patients. The patient characteristics are shown in **Table 1**. Twelve males (70.6%) and five females (29.4%) were included, with a median age of 63 years (range, 44–74 years). Most of the patients had IgG myeloma (10/17, 58.8%), the others had IgA (5/17, 29.4%), IgD (1/17, 5.9%) and λ light chain (1/17, 5.9%) myeloma. Thirteen of them (76.5%) were diagnosed with primary EMM, and four with secondary EMM (23.5%). There were 10 EM-B samples (58.8%) and 7 EM-E samples (41.2%), with available imaging data for extramedullary lesions in eight cases (**Supplementary Figures 1A, B** **)**. Nine patients had available cytogenetic information (9/17, 52.9%). The most frequent genetic abnormalities included deletion of chromosome 13q14, immunoglobulin heavy chain gene (IGH) translocations and 1q21 amplification.

**Table 1:** Patient Characteristics (*n* = 17).

No.	Gender	Age	Ig-type	BM %PC	BM Karyotype; FISH	EMM Lesions				
						Anatomic location	IHC staining		Incidence	Prior therapy
							CD56	Ki-67		
**EM-B**										
1	F	69	IgG κ	33%	*ND*	skull* ^†^	–	10%	primary	*NA*
2	F	50	IgG λ	80%	*ND*	clavicle*	+	20%	primary	*NA*
3	M	69	IgA λ	70%	46 XY; del13q14, transIgH	sternum*	+	20%	primary	*NA*
4	M	52	IgG κ	25%	*ND*	thoracic vertebra*	–	20%	primary	*NA*
5	M	69	IgG κ	50%	46 XY; Normal	thoracic vertebra*	–	15%	primary	*NA*
6	F	50	IgD κ	80%	46 XX; Normal	thoracic vertebra*	+	5%	primary	*NA*
7	M	66	IgG κ	30%	46 XY; del13q14, del14q32	thoracic vertebra*	+	30%	primary	*NA*
8	M	52	IgA κ	55%	46 XY; del13q14, amp1q21	thoracic vertebra*	–	20%	primary	*NA*
9	M	55	IgG κ	48%	CK; t(11;14)(q13;q32)	rib*	–	10%	primary	*NA*
10	F	51	IgG λ	90%	CK; *ND*	rib* ^†^	+	30%	primary	*NA*
**EM-E**										
11	M	63	IgA κ	80%	*ND*	mediastinum*	+	15%	primary	*NA*
12	M	74	IgA λ	50%	*ND*	prostate*^†^	–	80%	primary	*NA*
13	M	69	IgG λ	41%	*ND*	retroperitoneal lymph nodes*	+	15%	primary	*NA*
14	M	66	IgG λ	80%	CK; del13q14, transIgH, amp1q21	skin*, rib^†^	+	90%	secondary	BCD
15	F	63	IgA κ	25%	*ND*	skin*, pararenal space^†^, skull, pelvis, vertebra	+	80%	secondary	DVD
16	M	44	λ	90%	CK; amp1q21, t(14;16)(q32;q23)	skin*, liver, spleen, testicle	–	30%	secondary	CD, BD
17	M	63	IgG κ	80%	*ND*	spleen*, pleural effusion, sternum^†^	–	40%	secondary	VAD, BD, Rd

^*^EMM lesion for biopsy.

^†^EMM lesion with tumor mass ≥5 cm in diameter.

BM, bone marrow; PC, plasma cell; FISH, fluorescence in situ hybridization; EMM, extramedullary myeloma; EM-B, extramedullary-bone related; EM-E, extramedullary-extraosseous; IHC, immunohistochemistry; ND, not detected (met with a firm refusal); CK, complex karyotype; del, deletion; trans, translocation; IgH, immunoglobulin heavy chain; amp, amplification; NA, not available; BCD, bortezomib, cyclophosphamide, dexamethasone; DVD, liposomal doxorubicin, vincristine, dexamethasone; CD, cyclophosphamide, dexamethasone; BD, bortezomib, dexamethasone; VAD, doxorubicin, vincristine, dexamethasone; Rd, lenalidomide, dexamethasone.

**Figure 1:**
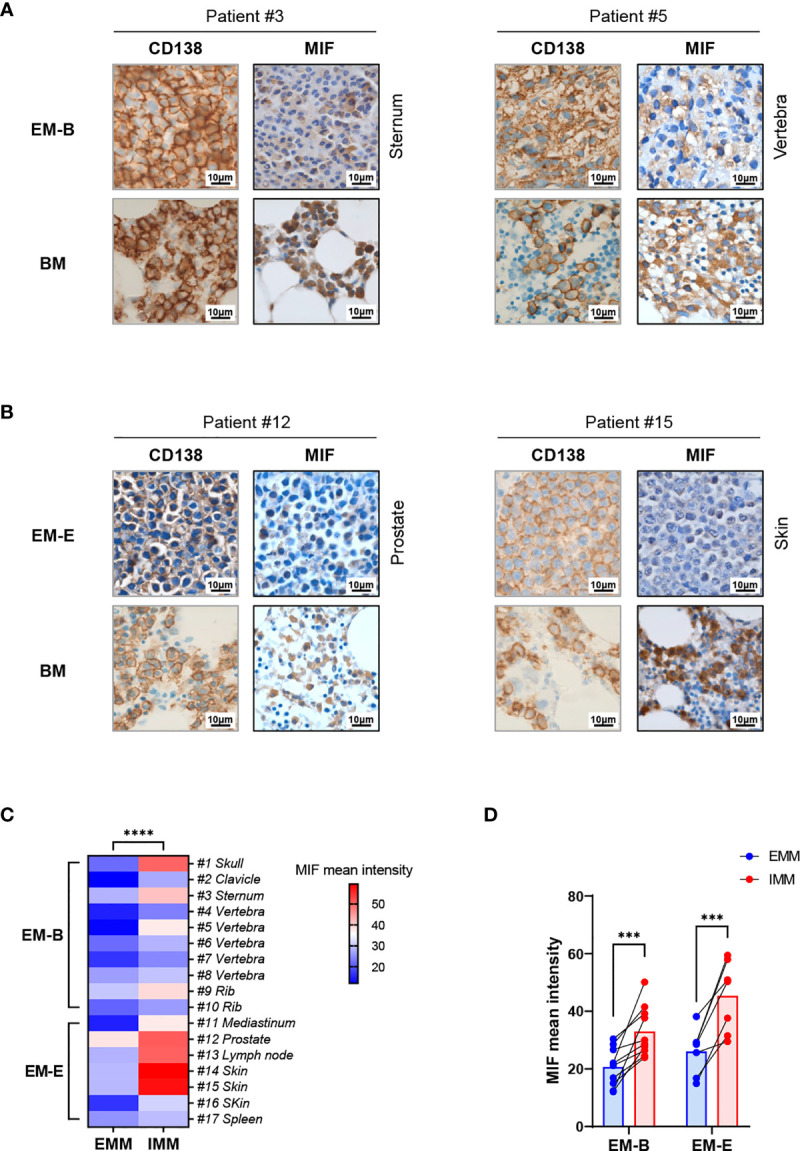
IHC staining showing MIF expression in EMM versus corresponding IMM. **(A, B)** IHC staining for CD138 and MIF in the paired samples from representative cases: the plasma cells highlighted by CD138 show higher MIF expression in IMM than in paired EMM, either for EM-B **(A)** or for EM-E (**B**; magnification 100×). **(C, D)** MIF intensity quantification based on wide-field IHC imaging indicates its intratumor heterogeneity; all EMM samples (n = 17) display downregulated MIF expression compared to the IMM counterparts. ****p < 0.0001, ***p < 0.001 (two-way ANOVA).

**Figure 2:**
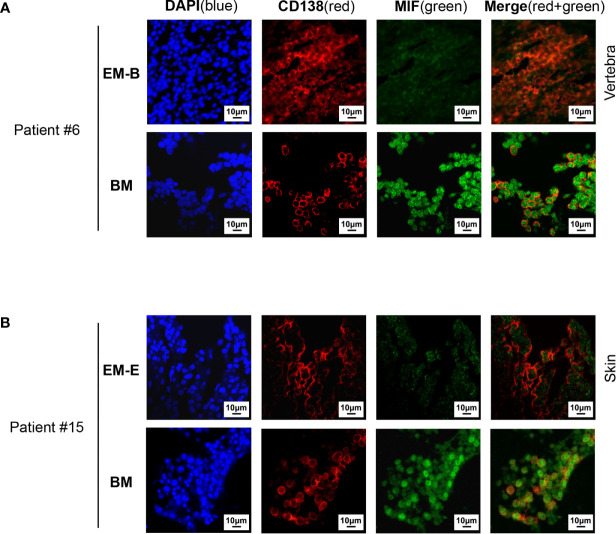
Immunofluorescence staining of MIF expression in EMM versus corresponding IMM. **(A, B)** Differential MIF expression in paired samples is also confirmed by opal multicolor staining in representative cases, including EM-B **(A)** and EM-E **(B)** (DAPI represents nuclear, red represents CD138, and green represents MIF; original magnification 40×).

Pathological analysis revealed higher MIF expression in IMM than in the paired EMM, as shown in the representative IHC staining in **Figures 1A, B** (all IHC data are available in **Supplementary Figure 2**). First, MM cells were positive for CD138 expression in all tumor samples (**Figures 1A, B** and **Supplementary Figure 2**). The invasive pattern in IMM varied from interstitial infiltration, focal aggregation to diffuse sheets. As for EMM, a diffuse or nest-like distribution of MM cells was more common. Second, MM cells from all tested samples generally showed cytoplasmic MIF expression. Nevertheless, differential expression of MIF was observed in EMM versus IMM (**Figures 1A, B** and **Supplementary Figure 2**). Quantification of MIF staining (**Supplementary Figure 3**) confirmed that MM-derived MIF expression was significantly higher in IMM than in EMM for the same patient (**Figures 1C, D**). In addition, we performed opal multicolor staining and immunofluorescence microscopy in selected paired FFPE samples to examine MIF expression. In agree with IHC data, lower MIF expression in EMM biopsies was observed in opal multicolor staining (**Figures 2A, B**). Overall, our data suggest that EMM clones tended to have down-regulated MIF expression compared with the corresponding IMM clones.

### Intratumor Heterogeneity of MIF Expression *In Vivo* in Myeloma Mouse Models

To further investigate the differential MIF expression in MM cells *in vivo*, immunodeficient mice were intravenously inoculated with two type of human MM cells. The primary BM MM cells were derived from a patient with advanced EM-E disease. The human MM cell line ARD was cell line-verified as single clonal PCs. MIF knockout ARD cells were used as a negative control for the animal study (**Supplementary Figure 4A**). All above cells showed consistent strong MIF expression *in vitro* (**Supplementary Figure 4B**). Compared with vehicle control, all the mice challenged with MM cells exhibited obvious morbidity as shown by significant weight loss, rough hair coat and limited mobility. Disease onset seemed to be earlier in ARD cell line-derived model than in the PDX model. *In vivo* BLI in mice revealed the formation of both IMM and EMM after inoculation with ARD or MIF^−/−^ ARD cells, the latter resulting more evident EMM (**Supplementary Figure 4C**), consistent with our previous findings (18). Tumor-bearing mice became moribund approximately 4–5 weeks after inoculation and thus to be euthanized (3–4 weeks after ARD cells injection, and 4–5 weeks after primary cells injection).

**Figure 3:**
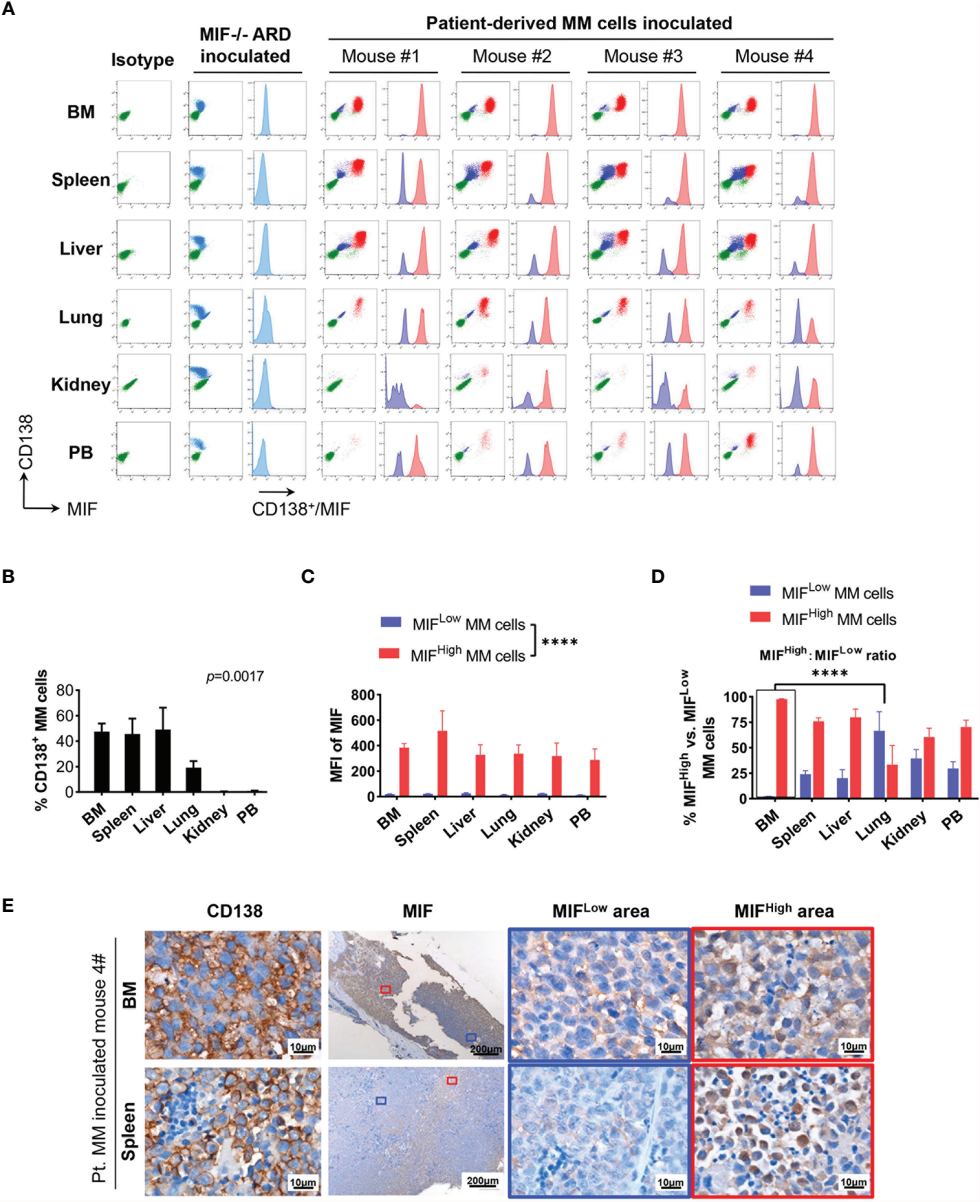
Identification of MIF^High^ and MIF^Low^ MM cells in patient-derived xenografts. **(A)** Patient #16-derived BM CD138^+^ cells were intravenously injected into B-NDG mice to generate a PDX model (n = 4). They were euthanized to collect fresh tissue cells for flow cytometry within four to five weeks after inoculation. Mice received MIF^−/−^ ARD cells were used as negative controls. Representative plots show the expression profiles of CD138 and MIF in isolated cells. Green represents CD138^−^ cells, namely non-MM cells; light blue represents MIF^−^ MM cells, dark blue represents MIF^Low^ MM cells, red represents MIF^High^ MM cells. **(B)** Percentage of infiltrating CD138^+^ MM cells varies considerably in collected samples (n = 4). *P* = 0.0017 (two-way ANOVA). **(C)** Compare mean fluorescence intensity (MFI) of MIF between MIF^High^ and MIF^Low^ populations in diverse samples. The mean value ± standard deviation is 362.5 ± 81.8 and 19.5 ± 5.5 respectively. ****p < 0.0001 (two-way ANOVA). **(D)** Ratio of MIF^High^/MIF^Low^ MM cells is notably higher in BM compared to extramedullary samples. ****p < 0.0001 (student’s *t*-test). **(E)** Representative IHC staining images of paired FFPE samples show sheets of MIF^Low^ CD138^+^ cells surrounded by MIF^High^ ones, and more evident loss of MIF expression in EMM (spleen) than in IMM (the blue and red boxes indicate representative areas of MIF^Low^ and MIF^High^ MM cells, respectively).

**Figure 4:**
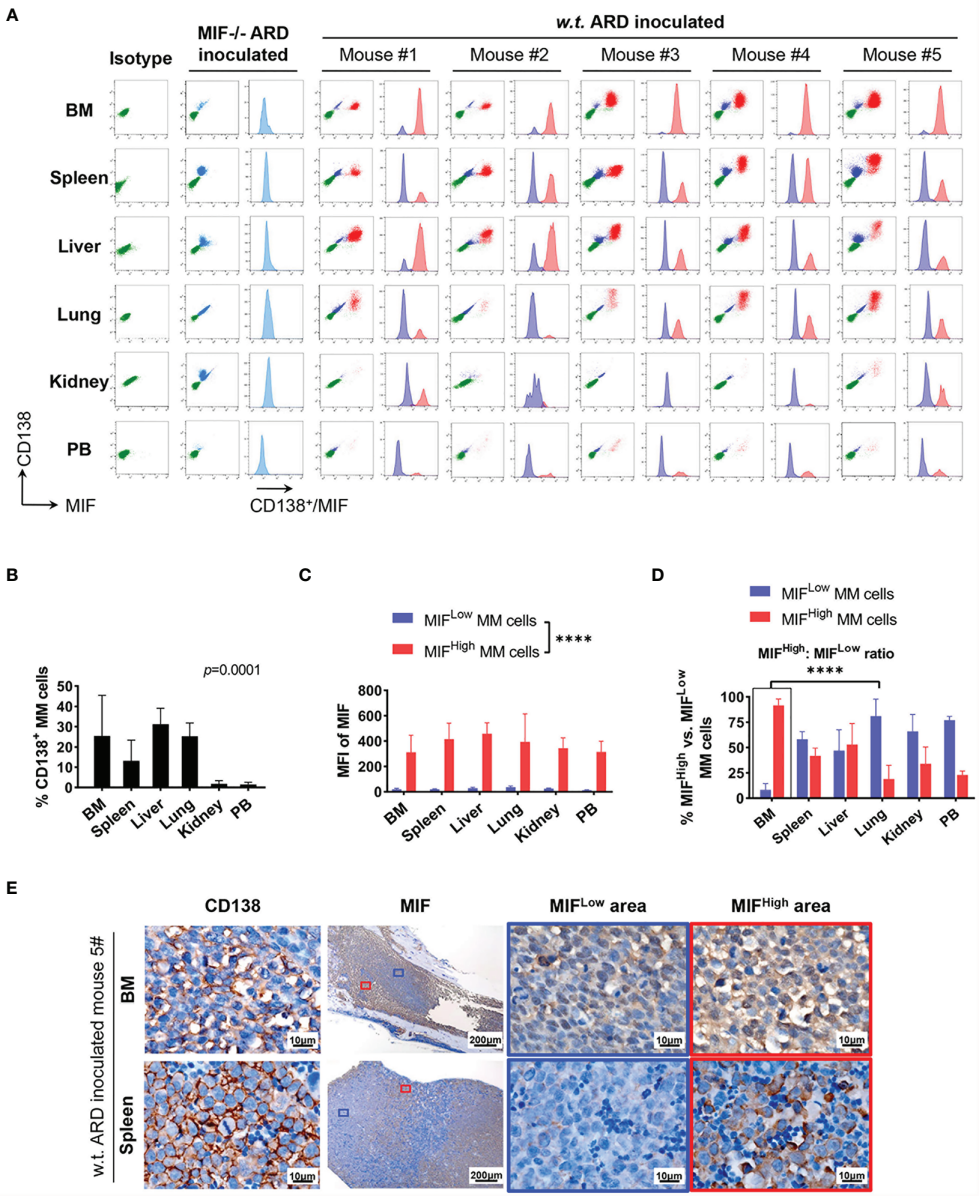
Identification of MIF^High^ and MIF^Low^ MM cells in ARD cell line-derived xenografts. **(A)** B-NDG mice were intravenously injected with ARD cell line to establish another xenograft model (n = 5). Control mice received vehicle (PBS) or MIF^−/−^ ARD cells. Three to four weeks after inoculation, samples were processed in the same way as described in the PDX model. Representative flow cytometry plots show MIF and CD138 expression in IMM and EMM (green, CD138^-^ cells, namely non-MM cells; light blue, MIF^-^ MM cells; dark blue, MIF^Low^ MM cells; red, MIF^High^ MM cells). **(B)** CD138^+^ cells (ARD cells) infiltrate all tested samples with varying degrees. *P* = 0.0001 (two-way ANOVA). **(C)** MFI of MIF differs greatly between MIF^High^ and MIF^Low^ MM cells in diverse samples, with 373.4 ± 59.3 (MIF^High^) versus 24.0 ± 8.1 (MIF^Low^). ****p < 0.0001 (two-way ANOVA). **(D)** Significantly higher ratio of MIF^High^/MIF^Low^ MM cells in BM versus extramedullary samples. ****p < 0.0001 (student’s *t*-test). **(E)** MIF^High^ and MIF^Low^ ARD cells *in vivo* are also identified by IHC. Representative images show their coexistence and more evident loss of MIF expression in spleen than in paired BM (the blue and red boxes indicate representative areas of MIF^Low^ and MIF^High^ MM cells, respectively).

Cells isolated from BM, visceral organs, and peripheral blood, were analyzed using flow cytometry for MIF expression in MM cells (CD138^+^ cells) *in vivo*. MM cells were identified in all tested samples with varied percentages, distributing mostly in BM, spleen, liver and lung (**Figures 3A, B**, **4A, B**). In MIF^−/−^ ARD inoculated mouse, the MM cells showed no MIF expression (**Figures 3A**, **4A**). Both patient primary MM cells and w.t. ARD cells exhibited two populations with differential MIF expression *in vivo*: MIF^High^ versus MIF^Low^. Mean fluorescence intensity (MFI) of MIF in MIF^High^ cells was more than 10 folds higher than that in MIF^Low^ cells (**Figures 3C**, **4C**). Of note, the relative proportion of MIF^High^ and MIF^Low^ MM cells was quite different between IMM and EMM samples. In general, IMM was found to have a significantly higher ratio of MIF^High^/MIF^Low^ MM cells compared to EMM: MM cells in BM were dominantly a MIF^High^ population, while in extramedullary organs or peripheral blood, the percentages of MIF^High^ cells decreased, and the MIF^Low^ population became remarkable **(** **Figures 3D**, **4D**). In the PDX scenario, MIF^High^ cells still constituted the majority of EMM, except the lung lesions (**Figure 3D**). As to the ARD model, MIF^High^ cells turned to be overwhelmed by MIF^Low^ ones in most EMM samples (**Figure 4D**). In addition, IHC staining for CD138 and MIF were performed in BM and spleen samples from tumor-bearing mice. The infiltrating cells positive for CD138 were a mixture of MIF^High^ and MIF^Low^ ones, with more evident loss of MIF expression in spleen versus BM (**Figures 3E**, **4E**).

To summarize, based on the above animal work, we found that a portion of MM cells lost their MIF expression (MIF^Low^) *in vivo*. IMM was dominated by the MIF^High^ MM cells. In EMM, by contrast, the expansion of MIF^High^ cells was accompanied by a notable MIF^Low^ population, which would attenuate the overall MIF expression. Somehow, those findings from the animal models might be consistent with our data of paired patient samples. Since a single clonal cell line was used in our model and the establishment of an MM-bearing model took only 3–4 weeks, it is not likely that the heterogeneity of MIF expression in MM cells was caused by genetic alterations.

### Single-Cell Transcriptome Study Reveals the Molecular Characteristics of Heterogenous Myeloma Subpopulations

Although MIF^Low^ MM cells *in vivo* were phenotypically distinct from MIF^High^ ones, physically separating them from each other for molecular study was not feasible because the permeabilization procedure of MIF staining would damage the nucleic acids in cells. Based on our previous findings, we hypothesized that the MM cells *in vivo* might have differential levels of MIF transcripts. Therefore, total CD138^+^ cells sorted from splenic cells in an ARD myeloma-bearing mouse (**Supplementary Figure 5A**) were subjected to single-cell RNA sequencing (scRNA-seq).

**Figure 5:**
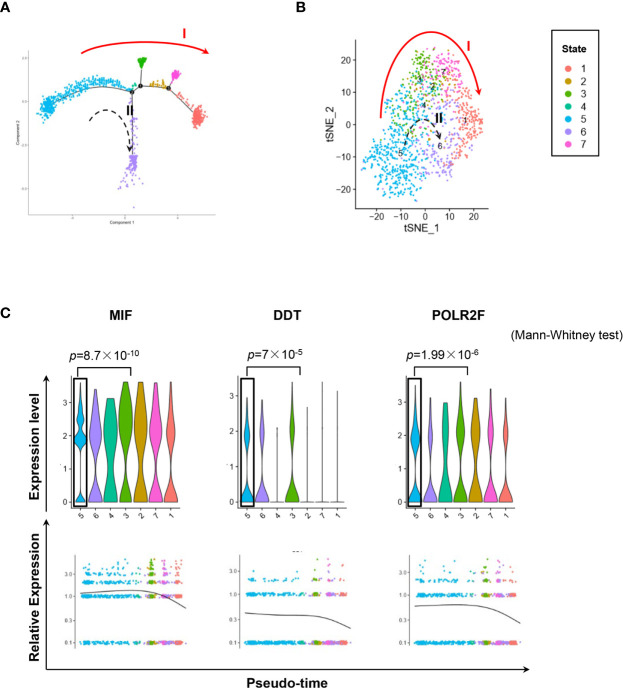
Single-cell transcriptomics exploring the trajectories of MIF^High^ to MIF^Low^ MM cells. **(A)** Sc-RNA seq explored the transcriptomic profiles associated with the development of MM cells in extramedullary microenvironment. MM cells labeled with the anti-human CD138 antibody were separated by flow cytometry from the spleen of an ARD MM-bearing mouse. A total of 1,424 extramedullary MM cells were plotted. The branching tree of MM cell trajectories define seven color-coded cell clusters or states: putatively developing from cluster #5 (initial state), proceeding along with several unstable intermediate states (clusters #3, #2 and #7, or cluster #4), and finally reaching cluster #1 or #6 (end state), as indicated by the red solid arrow (trajectory I) and black dotted arrow (trajectory II, probably transitional), respectively. **(B)** tSNE plots show seven clusters. Arrows I and II indicate the two putative cell-state transition trajectories, respectively. **(C)** Cluster #5 could reasonably be the initial state, since it has significantly higher expressions of MIF and two co-expressed genes DDT and POLR2F, as shown in the violin plots and scatter plots of pseudotime estimation. Likewise, the putative end state, especially cluster #1, has the least expression of MIF, DDT and POLR2F.

**Figure 6:**
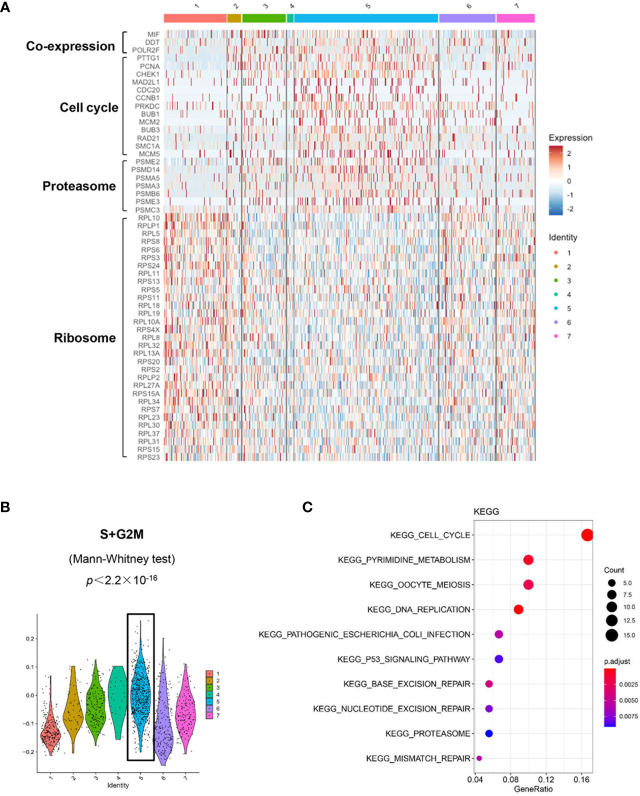
Distinct transcriptomic profiles of MM cells with differential MIF expression. **(A)** A heatmap of marker genes reveals distinct transcriptomic profiles of the MIF^High^ initial state (cluster #5) versus the MIF^Low^ end state (referring to cluster #1; cluster #6, lack of obvious gene expression signature, might actually be transitional). **(B)** Active proliferation is found in cluster #5 with a significantly higher score of S and G2/M-phase. **(C)** KEGG enrichment analysis indicates some important pathways, such as cell cycle, proteasome and p53 signaling pathway in cluster #5.

We used the DropSeq technique to investigate the single-cell transcriptomic profiles of the extramedullary plasma cells harvested from an ARD myeloma-bearing mouse. Given the lineage connectivity of these xenografted cells, we plotted the putative developmental trajectory using Monocle2 software. The software color-coded seven cellular states using the default parameters **(** **Figures 5A, B**). The violin plots showed significantly higher expression of MIF and two other co-expressed genes D-Dopachrome Tautomerase (DDT) and RNA Polymerase II Subunit F (POLR2F) in cluster #5, the co-expressions were validated in BM MM cells from multiple patient datasets (GSE26760, GSE19784, GSE9782) (26–28), which suggested cluster #5 was likely the initial state of these plasma cells (**Figure 5C** and **Supplementary Figure 5B**). Using the ‘FindMarkers’ function of Seurat, we further examined the signature genes of the clusters for state #5, the putative initial points of the cell lineage, as well as states #6 and #1, two putative end states of the cell lineage (**Figure 6A**). We carefully examined the marker genes using functional enrichments and had some interesting hits. The initial state, #5 cluster (MIF^High^ subpopulation), showed a significantly higher score of S and G2/M-phase (**Figure 6B**) and enriched the cell cycles, p53 signaling pathway, proteasome, etc. (**Figure 6C**). This finding suggests that the MIF^High^ feature is likely associated with a highly proliferative cell state. For the two end states of the trajectory, compared to cluster #5 of the initial state, cluster #1(MIF^Low^ subpopulation) gave many ribosome RNA signals, while cluster #6 gave no obvious signature expression, possibly indicating a transitional state (**Figure 6A**).

## Discussion

The presence of extramedullary myeloma, namely EMM, usually portends inferior outcomes for MM patients, even in the era of novel agents and stem cell transplantation (2, 29–31). Driven by multiple genetic and microenvironmental mechanisms, certain MM subclones are predisposed to survive outside the BM niche (5, 32). The molecular basis underlying EMM is still elusive. Although EMM typically results from contiguous or hematogenous spread of the intramedullary counterpart, EMM has its own distinctive biological features (1, 5). Here we proved the differential MIF expression between the matched IMM and EMM, both in patient samples and xenograft models.

The pleotropic biological activities of MIF have been characterized since 1960s (33–35). It is highlighted that MIF promotes progress and metastasis of various solid tumors, *via* activating downstream proliferation and anti-apoptotic signaling, regulating VEGF-mediated angiogenesis, inhibiting tumor suppressor P53, inducing immunosuppressive microenvironment and so on (36–43). Pathogenic role of MIF has also been identified in MM, and its expression level may serve as a surrogate for disease progression and prognosis (18, 34, 44–46). Recently, Wang et al. reported that MIF was implicated in proteasome inhibitor resistance by maintaining superoxide dismutase 1 (SOD1) activity and mitochondrial function (44). In that sense, MIF^High^ MM cells seem to be associated with more aggressive tumor behavior. However, MIF itself is an important chemokine favoring MM BM homing as we previously described (18). MIF^Low^ MM cells, generated by shRNA viral infection, would more easily egress from BM and form EMM in mice, due to impaired adhesion to the BM niche (18). Therefore, we assumed that MIF^Low^ clones, unlike in many solid tumors, may also implicated in MM extramedullary metastasis.

This study revealed that IMM was overwhelmed by MIF^High^ MM cells, while EMM showed a remarkably increased ratio of MIF^Low^ MM cells. This finding provided more evidence to suggest the link between decreased MIF and extramedullary colonization of MM cells. The differential dominance of the MIF^High^ clone in IMM versus EMM also indicated that the BM microenvironment had consistent selection pressure on MM cells to maintain the MIF^High^ phenotype. Predominance of MIF^Low^ MM cells in ARD-derived EMM was more obvious, not completely consistent with that in PDX scenario. It’s probably due to the biological difference between the two kinds of inoculated cells. Most noteworthy, ARD-derived xenograft and patient-derived xenograft, representing genetic homogeneity and heterogeneity respectively, could produce similar phenotypes in the short term after engraftment. Therefore, we inferred that an inheritable mechanism, likely involving some epigenetic changes rather than acquired genetic events, might govern the stable regulation of differential MIF expression.

Based on our ARD cell line-derived xenograft model, a further scRNA-seq was applied to explore the MIF-specific gene expression profiles in EMM. Our pseudotime analysis showed the consecutive alteration of MIF and its validated co-expressed genes along the developmental trajectory of extramedullary MM cells. Those transcriptional changes of MIF would be part of the reason for its differential expressions at the protein level. Furthermore, distinct transcriptomic profiles were revealed in the MIF^High^ and MIF^Low^ clusters, respectively. For one thing, the MIF^High^ cluster featured higher proliferation, while the MIF^Low^ cluster seemed much more quiescent. Previous *in vitro* studies by Joseph et al. demonstrated that mature CD138^+^ MM cells, being highly proliferative and chemosensitve, secreted more MIF than their quiescent, chemoresistant CD138^-^ progenitors, and addition of MIF inhibitor or recombinant MIF factor would promote the bidirectional interconversion (47). Specifically, our data suggested the *in vivo* correlation between MIF gene expression and MM cells proliferation, although no significant difference was found in CD138 expression between the two clusters. For another, the MIF^High^ cluster enriched the cell cycle, proteasome and P53 pathways, while the MIF^Low^ one harbored impressively abundant ribosomal protein genes. In a sense, this part of our data was consistent with the MIF-dependent tumor-promoting phenotypes reported in literature (37, 43, 44, 47, 48).Those differentially expressed marker genes between the two clusters also suggested potential upstream regulators of MIF transcription. To our knowledge, MIF transcription is mainly affected by various transcription factors, such as specificity protein 1 (SP1), cAMP response element binding protein (CREB), inverted CCAAT box binding protein of 90 kDa (ICBP90), and Hypoxia-induced factor 1α (HIF1α), and CATT repeats or G/C polymorphism at promoter region (49), the details in MM are as yet unknown.

It should be noted that the developmental trajectory of extramedullary MM cells was putative, dynamic, and even reversible. As MIF was highly expressed in MM cells before inoculation, we artificially defined MIF^High^ cluster as the initial state. In this case, adaptation to the microenvironment may account for the above findings. Another possibility is that MM cells homing to BM occasionally lose their MIF expression, and the MIF^Low^ clone would leave BM more easily to drive the extramedullary migration. Then MIF^Low^ cluster turned to be the alternative initial state.

In summary, our work identified the coexistence of MIF^High^ and MIF^Low^ MM cells, two distinct cell states with different proliferation ability and molecular profiles, and more obvious clonal expansion of the MIF^Low^ ones in EMM than that in IMM. The heterogeneous MIF expression *in vivo* was revealed at both the protein and mRNA level. We considered the loss of MIF expression in a portion of MM cells is mainly affected by microenvironment mechanisms instead of second genetic hits. Notably, our xenograft models could not entirely mimic the clinical course of EMM. Although the causal relationship of MIF^Low^ clone and EMM pathogenesis is still not definite, this study provides new insights into our understanding of MIF, a pathogenic effector and potential therapeutic target in MM. Nevertheless, questions remain about the exact regulation mechanisms, biological behavior and drug resistance properties of MM cells with differential MIF expression. We are conducting more studies to address those questions.

## Data Availability Statement

Our scRNA-seq data presented in this study can be found in online repositories. The names of the repository/repositories and accession number(s) can be found below: NCBI Gene Expression Omnibus (GEO; http://www.ncbi.nlm.nih.gov/geo/) under the accession number GSE175660.

## Ethics Statement

The studies involving human participants were reviewed and approved by the Ethical Committee of West China Hospital, Sichuan University, China. The patients/participants provided their written informed consent to participate in this study. The animal study was reviewed and approved by the the Animal Care and Use Committees of West China Hospital, Sichuan University.

## Author Contributions

YHZ designed and supervised the research, and prepared the manuscript. SZ provided the patients’ samples, reviewed the results of IHC staining, assisted with data interpretation. YMZ provided the biological insight from scRNA-seq data, and prepared the manuscript. YHZ, SZ, and YMZ contributed equally to this work as co-corresponding authors. JX performed the experiments, data analysis and prepared the manuscript. NY and WLin performed the extensive scRNA-seq data analyses and prepared the manuscript. JX and NY contributed equally as co-first authors to this manuscript. PZ conducted the MIF gene knock-out experiment. FW and JH assisted with research design, and provided critical suggestions. YC, HD, YY, and YG assisted with experimental work. The other authors provided the basic clinicopathological data. All authors contributed to the article and approved the submitted version.

## Funding

This work was supported by grants to YHZ from the National Natural Science Foundation of China (Nos. 81870157 and 82070219), Science and Technology Department of Sichuan Province (No. 2019YJ0028), and the Sichuan University Faculty Start Fund. Grants to JH from the National Natural Science Foundation of China (No. 81800207) and the Health Commission of Sichuan Province (No. 18PJ357). A grant to YQ from Science and Technology Department of Sichuan Province (No. 2018FZ0030).

## Conflict of Interest

The authors declare that the research was conducted in the absence of any commercial or financial relationships that could be construed as a potential conflict of interest.

## Supplementary Material

Supplementary Figure 1 Imagings of extramedullary myeloma lesions for biopsy in patients. The collected EMM tissue samples were bone-related (EM-B) or extraosseous (EM-E). Corresponding imaging data were available for 8 of them. Magnetic resonance imaging (MRI) or computed tomography (CT) showed the biopsied EM-B **(A)** and EM-E **(B)** lesions respectively (indicated by the red arrow).

Supplementary Figure 2 Differential MIF expression in paired IMM and EMM. Seventeen pairs of FFPE samples were stained for CD138 and MIF. In all tested slides, the infiltrating MM cells were specifically highlighted by membranous CD138 staining, and showed cytoplasmic positivity for MIF to varying degrees. Generally, IMM had higher MIF expression in comparison to the paired EMM.

Supplementary Figure 3 Quantification method of immunostain for MIF in paired patient samples. Quantitative analysis of MIF staining was as follows (showing a representative pair):wide-field images of BM and EM tissue slides were captured with the TissueFAXS system (TissueGnostics ), then StrataQuest software (TissueGnostics) was used to convert DAB channels of MIF staining into gray scale images and to evaluate the mean intensity of MIF in effective positive cells as dot plots show.

Supplementary Figure 4 Supplementary data of the animal experiment. Complete knockout of the MIF gene in ARD cells was verified by western blot **(A)**. Flow cytometry analysis showed the in vitro expression of CD138 and MIF in these three types of grafts: All of them were positive for CD138; MIF was homogenously over-expressed in both ARD cells and patient-derived primary BM MM cells, and totally absent in MIF-/-ARD cells **(B)**. Intravenous injection of human MM cells led to both intra- and extra-medullary lesions in immunodeficient mice. More serious EMM lesions were detected in the MIF-/- ARD group. The representative *in vivo* bioluminescent imagings were taken at two weeks after inoculation **(C)**.

Supplementary Figure 5 Supplementary data of the sc-RNAseq. Parallel flow cytometry analysis confirmed the differential expression of MIF protein in CD138+ cells subjected to sc-RNAseq **(A)**. Co-expression of DDT, POLR2F and MIF in patient MM cells from BM was validated in three microarray datasets: GSE26760, GSE19784, and GSE9782, by gene set enrichment analysis (GSEA) with Pearson correlation (R2, R-square) **(B)**.
