# Supplementary material for: Intratumor Heterogeneity of MIF Expression Correlates With Extramedullary Involvement of Multiple Myeloma
Source: Front Oncol. 2021 Jun 29;11:694331. doi: 10.3389/fonc.2021.694331 (PMC8276700; doi:10.3389/fonc.2021.694331)
Supplement: Supplementary Figure 1 — Imagings of extramedullary myeloma lesions for biopsy in patients. The collected EMM tissue samples were bone-related (EM-B) or extraosseous (EM-E). Corresponding imaging data were available for 8 of them. Magnetic resonance imaging (MRI) or computed tomography (CT) showed the biopsied EM-B (A) and EM-E (B) lesions respectively (indicated by the red arrow). [file DataSheet_1.pdf]

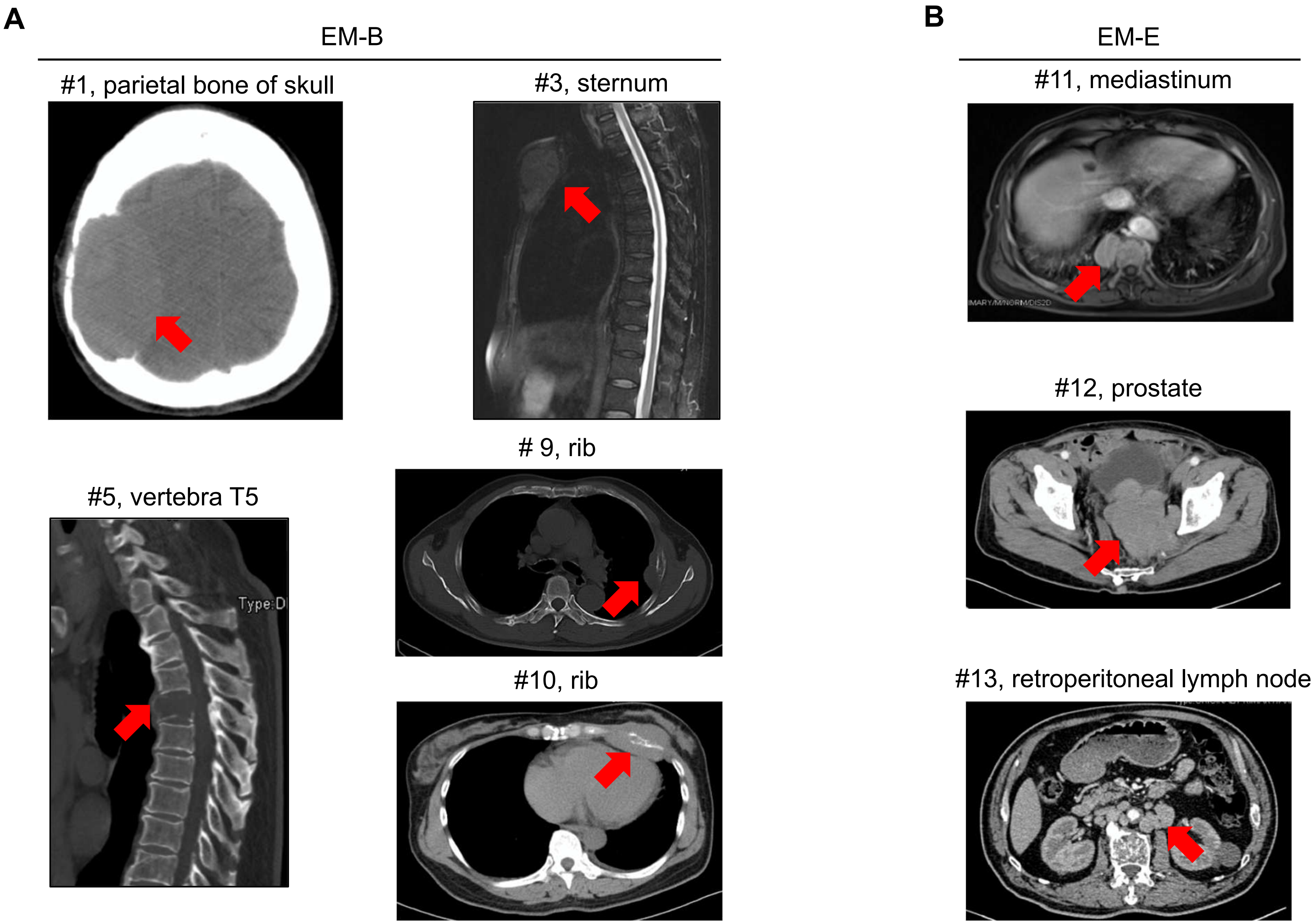

Fig S1. Imagings of extramedullary myeloma lesions for biopsy in patients

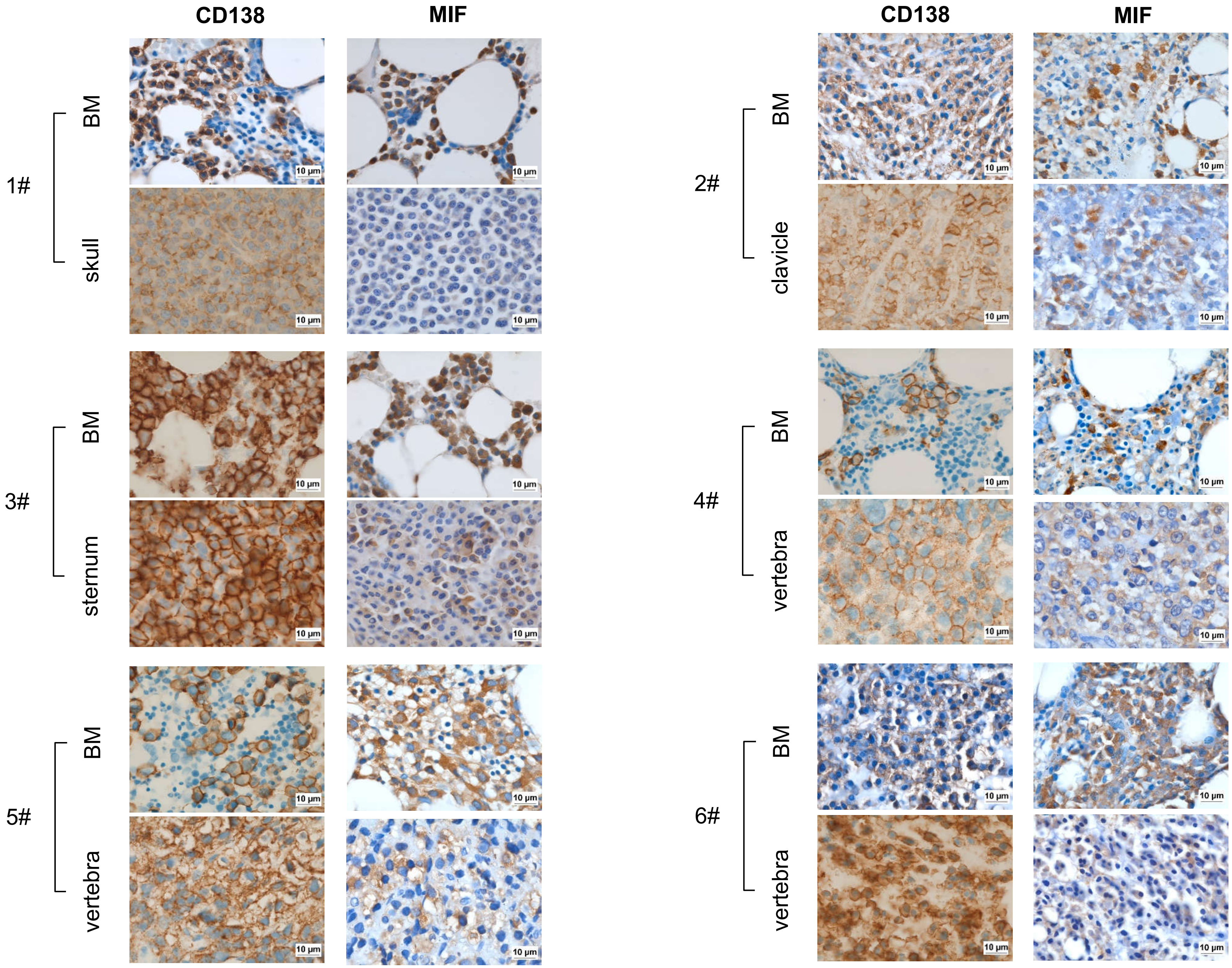
CD138
MIF
1#
BM
skull
2#
clavicle
3#
sternum
4#
vertebra
5#
6#
10 μm

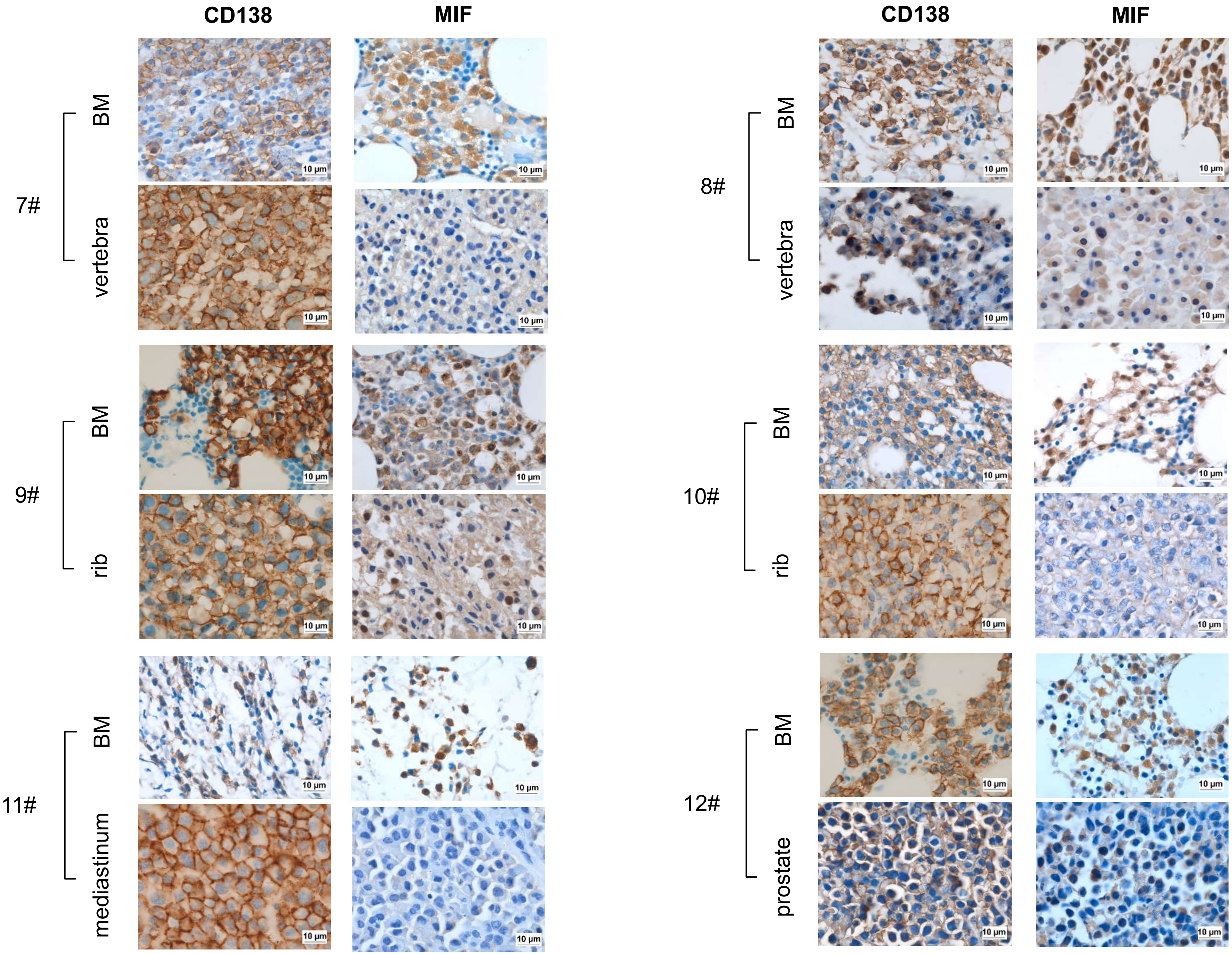
CD138
MIF
CD138
MIF
7#
BM
vertebra
8#
BM
vertebra
9#
BM
rib
10#
BM
rib
11#
BM
mediastinum
12#
BM
prostate
10 μm

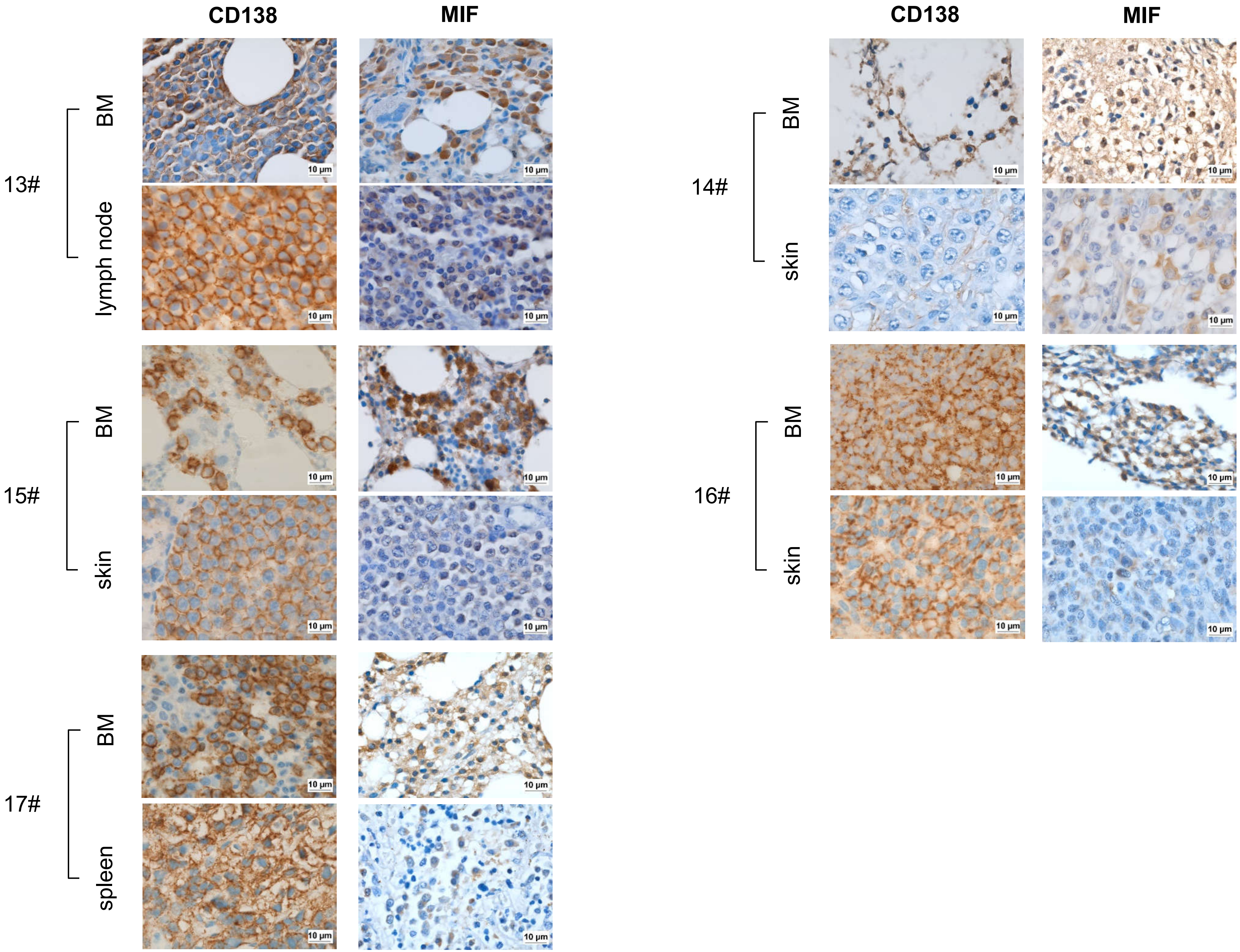

Fig S2. Differential MIF expression in paired IMM and EMM

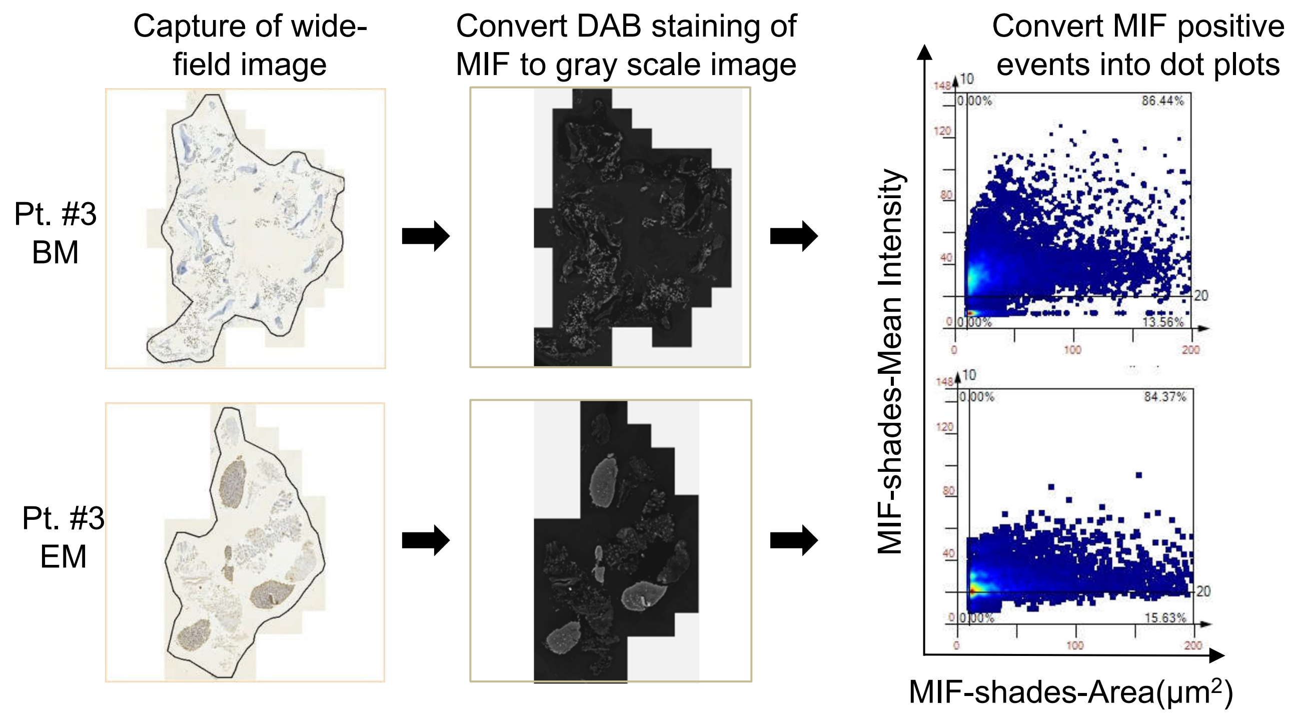

Fig S3. Quantification method of immunostain for MIF in paired patient samples

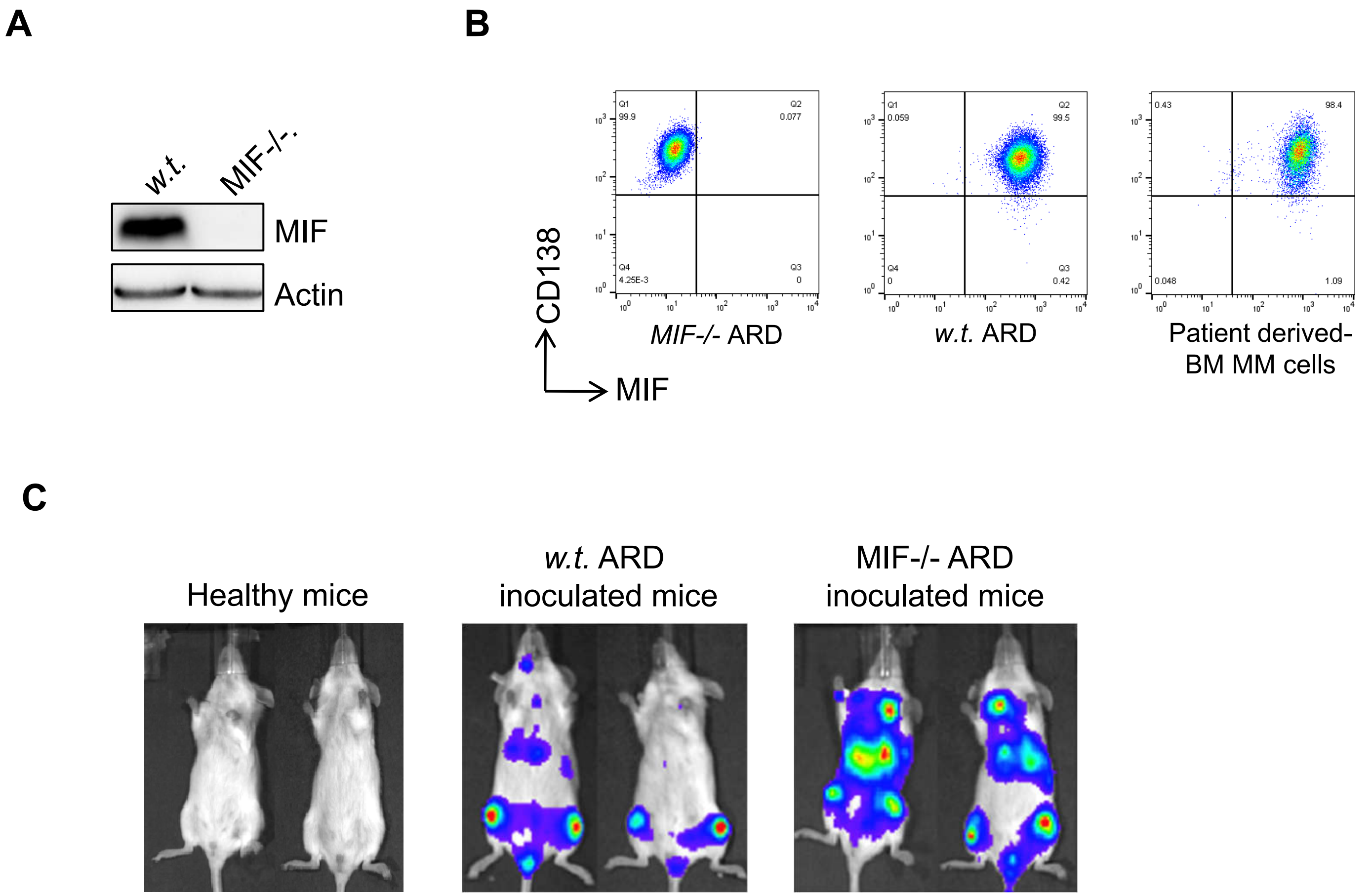

Fig S4. Supplementary data of the animal experiment

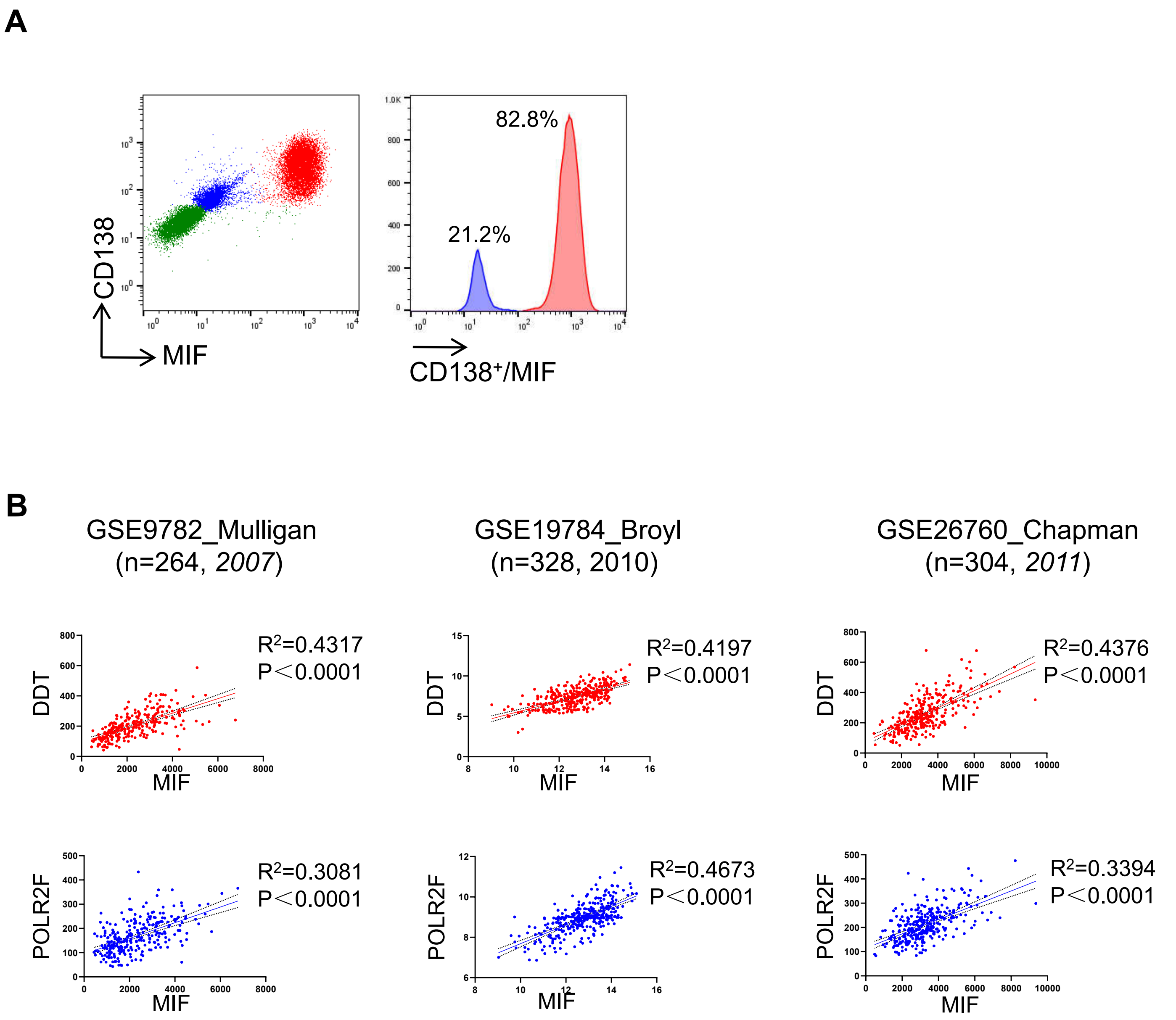

Fig S5. Supplementary data of the sc-RNAseq
